# Role of CCR2-Positive Macrophages in Pathological Ventricular Remodelling

**DOI:** 10.3390/biomedicines10030661

**Published:** 2022-03-12

**Authors:** Veera Ganesh Yerra, Andrew Advani

**Affiliations:** Keenan Research Centre for Biomedical Science, Li Ka Shing Knowledge Institute, St. Michael’s Hospital, Toronto, ON M5B 1T8, Canada; veeraganesh.yerra@unityhealth.to

**Keywords:** inflammation, heart failure, ventricular remodelling, macrophage, monocyte, CCR2, single-cell RNA sequencing, myocardial infarction, pressure overload

## Abstract

Even with recent advances in care, heart failure remains a major cause of morbidity and mortality, which urgently needs new treatments. One of the major antecedents of heart failure is pathological ventricular remodelling, the abnormal change in the size, shape, function or composition of the cardiac ventricles in response to load or injury. Accumulating immune cell subpopulations contribute to the change in cardiac cellular composition that occurs during ventricular remodelling, and these immune cells can facilitate heart failure development. Among cardiac immune cell subpopulations, macrophages that are recognized by their transcriptional or cell-surface expression of the chemokine receptor C-C chemokine receptor type 2 (CCR2), have emerged as playing an especially important role in adverse remodelling. Here, we assimilate the literature that has been generated over the past two decades describing the pathological roles that CCR2^+^ macrophages play in ventricular remodelling. The goal is to facilitate research and innovation efforts in heart failure therapeutics by drawing attention to the importance of studying the manner by which CCR2^+^ macrophages mediate their deleterious effects.

## 1. Introduction

Heart failure is the clinical manifestation of a heterogeneous group of conditions that often co-exist and that collectively cause impairment in the filling or emptying of the cardiac chambers. This heterogeneity in etiology poses problems for the discovery and development of new therapies that aim to improve heart failure outcomes. To circumvent some of these challenges, research and development efforts are often focused on common pathogenic mechanisms that cause heart failure development, irrespective of the underlying primary etiology. Pathological ventricular remodelling frequently precedes heart failure development. However, the cellular mechanisms that actually cause ventricular remodelling and lead to the clinical presentation of heart failure are incompletely understood. Immune cells have long been linked to pathological remodelling. In recent years, technological advances in genetic fate mapping and single-cell transcriptomics have revealed that immune cell populations in the heart are much more heterogeneous than was initially anticipated. Despite this heterogeneity, monocytes and macrophages that express C-C chemokine receptor type 2 (CCR2) on their surfaces have consistently been linked with adverse cardiovascular outcomes. Here, we summarize the evidence implicating CCR2^+^ monocyte-derived macrophages in pathological ventricular remodelling. In doing so, we hope to facilitate advances in heart failure therapeutics by improving accessibility to the field of cardiac immunology for those who may be new to it.

## 2. Heart Failure Is a Major Burden on Population Health

Heart failure is a problem that affects an estimated 64 million people worldwide [1]. In developed countries, between 1% and 2% of the population have been diagnosed with heart failure, and roughly a similar percentage of the population live with undiagnosed heart failure [2]. The morbidity and mortality associated with heart failure are substantial. For instance, heart failure is the leading primary diagnosis for hospitalization in the United States (U.S.) [3] and is responsible for up to 2% of all hospital admissions [4]. Furthermore, among persons hospitalized for heart failure, the five-year mortality rate is as high as 75–78% [5,6]. Over the past twenty years, the incidence of heart failure appears to have declined. In the Olmsted County cohort in the U.S., for example, the age- and sex-adjusted incidence of heart failure declined from 3.2 cases per 1000 person–years in 2000 to 2.2 cases per 1000 person–years in 2010, equating to a rate reduction of 37.5% [7]. However, with improvements in acute and chronic management, along with an aging population, the total number of cases of heart failure has actually risen, even in the face of this declining incidence. For example, it has been estimated that between 2002 and 2014, the absolute number of prevalent heart failure cases in the United Kingdom (U.K.) increased by 23% [8]. Heart failure also poses a substantial economic burden, with its direct and indirect costs in the U.S. being expected to reach USD 70 billion by 2030 [9]. In short, despite preventative and therapeutic advances, heart failure remains a major health threat and there remains a pressing need for innovative new treatments.

## 3. Heart Failure Is a Heterogeneous Condition

Heart failure has been defined as being “a complex clinical syndrome that results from any structural or functional impairment of ventricular filling or ejection of blood” [10]. Heart failure is highly heterogeneous, both in its presentation and in its etiology. Chronic heart failure is typically subdivided according to whether there is predominantly a problem with the ejection of blood (heart failure with reduced ejection fraction (ejection fraction (EF) < 40%); HFrEF) or a problem with ventricular filling (heart failure with preserved ejection fraction (EF ≥ 50%); HfpEF), with intermediate EF levels being categorized as heart failure with mid-range ejection fraction (HfmEF; 40% ≤ EF < 50%) [11]. Being a chronic stage of any disease that results in functional cardiac impairment, heart failure has a variety of causes that often co-exist [2] and is typically associated with the activation of neurohumoral pathways and sympathetic nervous system activation. It has been estimated that coronary artery disease (CAD) is the cause of approximately two-thirds of cases of heart failure [12]. Thus, one convenient way to categorize heart failure according to its etiology is to subdivide it into ischemic heart failure or non-ischemic heart failure, depending on the presence or absence of CAD. In practice, however, multiple conditions that each predispose to heart failure often occur in concert (e.g., CAD, hypertension and diabetes), meaning that, in many cases, it is not possible to attribute heart failure to a single etiology. Despite these disparate etiologies, a common pathophysiological process that often precedes heart failure development, and contributes to its development, is adverse ventricular remodelling. Accordingly, understanding the cellular and molecular processes that occur during pathological ventricular remodelling could open up new therapeutic avenues to prevent heart failure occurrence or slow its progression.

## 4. Ventricular Remodelling Is a Common Antecedent of Heart Failure

Cardiac remodelling refers to the change in the size, shape, function and/or composition of the heart in response to load or injury [13]. This remodelling can be physiological (e.g., in response to exercise training) or pathological (e.g., following MI or in response to a pressure overload caused by hypertension or obstructive aortic valve disease). The importance of remodelling in the pathogenesis of heart failure is most readily appreciated when considering the ventricular response to MI. In the initial phase after MI, tissue in the infarct zone goes through a process of cell death, inflammation and scar formation, which prevents ventricular rupture but subsequently leads to elongation and thinning of the ventricular wall [14,15]. This results in an increase in volume and pressure load placed on the non-infarcted myocardium which, in turn, induces cardiomyocyte hypertrophy and an increase in ventricular wall mass and chamber enlargement. These structural responses after MI cause a shift in the typical geometry of the ventricle from an elliptical configuration to a less efficient spherical configuration, which brings about a progressive diminution in ventricular performance and the clinical manifestation of heart failure [14,15]. Although pathological ventricular remodelling is commonly caused by MI, it can also occur in the absence of cardiomyocyte death and is also a common antecedent of non-ischemic heart failure. For example, hypertension accounts for approximately 10% of cases of heart failure [16]. Pressure overload, caused by hypertension (or aortic stenosis, for example) causes concentric ventricular remodelling that is characterized by the thickening of the ventricular wall and widening of cardiomyocytes. Volume overload, in contrast, causes eccentric ventricular remodelling and MI gives rise to mixed concentric and eccentric remodelling. Clinically, ventricular remodelling in HFrEF is commonly detected by observation of a reduction in EF and an increase in left ventricular (LV) end-systolic volume (LVESV) and LV end-diastolic volume (LVEDV), which can be determined non-invasively by echocardiography [14]. While concentric remodelling and increased ventricular wall thickness are common in HFpEF, LV geometry is more heterogeneous, and can also be unchanged or associated with eccentric remodelling [17].

A large body of evidence exists highlighting the association between adverse ventricular remodelling and cardiovascular outcomes. For example, in the Valsartan in Acute Myocardial Infarction (VALIANT) study, baseline EF, LVESV and LVEDV were each independent predictors of total mortality, death or hospitalization for heart failure or death or any cardiovascular event (heart failure, MI, stroke, resuscitated sudden death) [18]. Furthermore, numerous studies have shown that interventions that improve ventricular remodelling also improve heart failure outcomes [14]. For HFrEF, this is the case for beta-blockers [19,20], angiotensin-converting enzyme (ACE) inhibitors [21,22], angiotensin II receptor blockers [23,24], angiotensin receptor II—neprilysin inhibition (ARNI) [25,26] and cardiac resynchronization therapy (CRT) [27,28]. Recent paradigm-altering studies of sodium-glucose cotransporter-2 (SGLT2) inhibition have gone on to extend the relationship between change in ventricular remodelling and heart failure outcomes to also include patients with HFpEF. In the EMPA-HEART study, for example, treatment of participants with Type 2 diabetes and known CAD with the SGLT2 inhibitor empagliflozin was associated with a significant reduction in LV mass, as determined by cardiac magnetic resonance imaging [29]. In the Empagliflozin Outcome Trial in Patients with Chronic Heart Failure and Reduced Ejection Fraction (EMPEROR-Reduced) and Empagliflozin Outcome Trial in Patients with Chronic Heart Failure and Preserved Ejection Fraction (EMPEROR-Preserved) studies, empagliflozin improved the primary endpoint of cardiovascular death or hospitalization for heart failure in participants with HFrEF, HFmEF or HFpEF [30,31]. Despite these therapeutic advances, the prognosis for heart failure remains poor. In EMPEROR-Preserved, the primary outcome still occurred in 13.8% of participants treated with empagliflozin over a median follow-up period of 26.2 months [31]. In EMPEROR-Reduced, the primary endpoint occurred in 19.4% of empagliflozin-treated participants over a median follow-up of 16 months [30]. A better understanding of the cellular basis of ventricular remodelling could open up future innovative treatments to further improve outcomes for people living with, or at risk of developing, heart failure.

## 5. Monocyte-Derived Macrophages Contribute to Adverse Ventricular Remodelling in Humans

In addition to changes in the size and shape of the ventricular walls and the size and shape of cardiomyocytes, a number of other cellular, molecular, transcriptional, electrophysiological and neurohormonal changes accompany ventricular remodelling and play important roles in the progression to heart failure. These include myocyte cell death by either apoptosis or necrosis, activation and proliferation of cardiac fibroblasts, endothelial to mesenchymal transition, deposition of fibrotic collagenous matrix within the interstitium, capillary rarefaction, activation of proinflammatory intracellular pathways, renin angiotensin aldosterone system (RAAS) activation, perturbations in beta-adrenergic signalling, excitation–contraction coupling, mitochondrial oxidative metabolism and inflammatory cell recruitment [32].

Monocyte infiltration of the myocardium has been linked to ventricular remodelling for over 20 years [33]. Peripheral monocytosis occurs 2–3 days after MI and, in patients with MI, correlates positively with LVEDV and negatively with EF, acting as an independent predictor of pump failure, LV aneurysm and future cardiac events [34]. Similarly, an accumulation of macrophages has been reported in LV myocardial biopsies of patients with hypertension and HFpEF [35]. These studies, and many other classical experiments like them, point to the importance of mobilization of monocytes during ventricular remodelling, recruitment of these cells to the heart, differentiation to macrophages and a deleterious effect of these accumulated pro-inflammatory macrophages on heart function.

## 6. CCR2^+^ Monocyte-Derived Macrophages Have Emerged as Key Effectors of the Inflammatory Response to Myocardial Injury

In 2000, Mills and co-workers first proposed their heuristic through which macrophages may be classified as M1 (ostensibly pro-inflammatory) or M2 (ostensibly reparatory), based on the functional response of the macrophages to T lymphocyte cytokines [36]. In their original description, however, the authors stressed that the dichotomous M1/M2 classification of macrophages “…while useful for conceptualizing immune responses, certainly could be an oversimplification… Instead, there may be a continuum of phenotypes between M-1 and M-2 macrophages” [36]. As foreseen by the authors in their original report, this has indeed proven to be the case. Advances in technologies such as genetic fate mapping, single-cell RNA sequencing, cytometry by time of flight (CyTOF) and non-invasive hyperspectral imaging have confirmed that monocyte/macrophage populations, particularly macrophage populations in the heart, are far more heterogeneous and more plastic than accounted for by the classical M1/M2 paradigm. Nevertheless, the expression of marker genes and/or particular cell surface markers has enabled the categorization of monocytes and macrophages into subgroups with distinct origins and functional properties.

Circulating monocytes can be divided into subsets based upon their expression of chemokine receptors and cell-surface markers. In mice, monocytes can be subdivided according to their expression of Ly6C, a member of the lymphocyte antigen-6 superfamily and a glycosylphosphatidylinositol (GPI)-anchored protein [37,38]. Ly6Chi monocytes are classically considered as inflammatory and give rise to M1-like macrophages [39]. They express high levels of the chemokine receptor, CCR2 and low levels of the chemokine receptor, C-X3-C motif chemokine receptor 1 (CX3CR1) [40]. Ly6Clo monocytes are considered to be non-classical [39]. They express high levels of CX3CR1 and low levels of CCR2. Ly6Clo monocytes are lower in abundance than Ly6Chi monocytes and they are patrolling cells that can promote angiogenesis and tissue remodelling [41]. Ly6Chi monocytes derive from bone marrow hematopoietic progenitors and Ly6Clo monocytes arise from Ly6Chi monocytes by conversion [39]. In 2007, Nahrendorf and co-workers demonstrated that Ly6Chi and Ly6Clo monocytes are sequentially recruited to the heart after MI via CCR2 and CX3CR1, respectively [42]. Human monocytes are subdivided into three categories according to their surface expression of CD14 and CD16: CD14^++^CD16^−^ (classical), CD14^++^CD16^+^ (intermediate) and CD14^+^CD16^++^ (non-classical). CD14^++^CD16^−^ monocytes are similar to Ly6Chi monocytes, are pro-inflammatory and express high levels of CCR2 [40]. The half-life of inflammatory Ly6Chi monocytes is very short (~8 h), and during this period the cells either convert into long-lived patrolling Ly6Clo monocytes in the blood vessels or they enter non-lymphoid organs and mature into macrophages or dendritic cells [41]. Only a small subset of monocytes that enter the tissue remain in their original state without being converted to macrophages or dendritic cells, and these are termed “tissue monocytes” [41].

**Figure 1:**
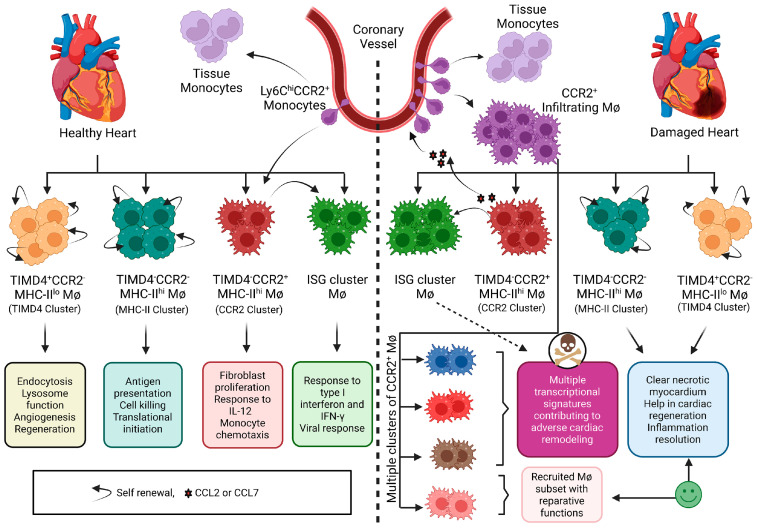
Illustrating the contemporary perspective on cardiac macrophage heterogeneity under steady state and disease condition, based on findings published and discussed in [43,46]. By single-cell RNA sequencing, under steady-state conditions, the healthy adult mouse heart contains four clusters of cardiac macrophages (TIMD4, MHC-II, CCR2 and ISG clusters). Both TIMD4 and MHC-II clusters are maintained by in situ, proliferation-based self-renewal. The CCR2 cluster is derived from circulating monocytes. The ISG cluster is derived and maintained by the CCR2 cluster rather than directly from monocytes. Some Ly6Chi monocytes that enter the tissue remain as “tissue monocytes” without converting to macrophages or dendritic cells. Under disease conditions, large numbers of circulating monocytes infiltrate the myocardium under the influence of chemokines secreted from tissue resident CCR2^+^ macrophages and other cells. CCR2^+^ infiltrating macrophages derived from circulating monocytes acquire different transcriptional active states and contribute to adverse cardiac remodelling changes. Some recruited macrophages may acquire states that help in the resolution of inflammation and repair of tissue. The TIMD4 cluster and MHC-II cluster resident macrophages exert reparative functions to heal cardiac damage and help in tissue regeneration. The ISG cluster is also expanded after injury and its function and contribution to adverse cardiac remodelling is uncertain. The change in the number of cells from each cluster in the disease state indicates their increase or decrease with respect to the steady state. Abbreviations: TIMD4 = T cell immunoglobulin and mucin domain containing 4, CCR2 = C-C chemokine receptor type 2, MHC-II = major histocompatibility complex class II, ISG = interferon stimulated gene, Ly6C = lymphocyte antigen 6 complex, locus C1, Mφ = macrophage, CCL2 = C-C motif chemokine ligand 2, CC7 = C-C motif chemokine ligand 7.

Cardiac macrophages are far more transcriptionally heterogeneous than suggested by the classical M1/M2 paradigm. Readers are referred to an excellent recent review on this heterogeneity [43] and the associated source publications supporting it. As may be expected, given their heterogeneity, there are some differences in the monocyte and macrophage subpopulations that have been described in various reports, which are likely explained by the different models that were studied and the experimental methods and data-processing strategies that were adopted. Despite these differences, the presence or absence of CCR2 has consistently stood out as being a robust marker of macrophage origin and phenotype. For instance, in 2014, genetic fate mapping studies enabled Epelman et al. to demonstrate that CCR2 expression distinguishes monocyte-derived cardiac macrophages from those that are embryonic in origin [44]. The same year, through flow cytometry and genetic lineage tracing, investigators established that the adult heart contains two resident macrophage populations (MHC-IIloCCR2^−^ and MHC-IIhiCCR2^−^), one monocyte-derived macrophage population (MHC-IIhiCCR2^+^) and one monocyte population (MHC-IIloCCR2^+^), and that the injured adult heart selectively recruits monocytes and MHC-IIhiCCR2^+^ monocyte-derived macrophages [45]. More recently, following up on these studies, investigators combined experiments using genetic fate mapping, long-term parabiosis and single-cell RNA sequencing to refine this subcategorization into four subsets of cardiac monocytes/macrophages: a CCR2^−^ subset, maintained independently of monocytes (TIMD4+LYVE1+MHC-IIloCCR2^−^); a CCR2^−^ subset that is partially replaced by monocytes (TIMD4-LYVE1-MHC-IIhiCCR2^−^) and two CCR2^+^MHC-IIhi subsets that are fully replaced by monocytes, whereas these subsets diversified significantly after ischemic injury [46]. Figure 1 illustrates this contemporary view of cardiac macrophage subpopulations. In a separate single-cell RNA sequencing experiment of pressure overloaded mouse hearts, two transcriptionally distinct CCR2^−^ monocyte/macrophage clusters were identified, one representing resident pro-repair macrophages and the other CCR2^−^ cluster likely representing M1-like phagocytic macrophages/monocytes [47].

Simplistically, despite some variations in the manner of their description in the literature, tissue macrophages in the adult heart can be subcategorized according to their origin (either embryonic-derived or monocyte-derived) and their expression (or lack of expression) of the chemokine receptor, CCR2. The adult myocardium contains CCR2^−^ macrophages, CCR2^+^ monocytes and CCR2^+^ macrophages [48]. CCR2^−^ cardiac macrophages are embryonic in origin, arising from the yolk sac or fetal liver hematopoietic progenitors. They are long-lived and replenished locally and they largely function in a reparative capacity [44,45,46,48,49]. CCR2^+^ cardiac macrophages are derived from hematopoietic progenitors; they are maintained and expanded through monocyte recruitment and local proliferation; they produce inflammatory cytokines, facilitate neutrophil and monocyte recruitment and contribute to cardiac oxidative stress, thus augmenting myocardial injury and facilitating adverse remodelling [44,45,46,48,49,50,51,52,53].

Although the field is in its comparative infancy, evidence is beginning to indicate that resident CCR2^−^ macrophages limit adverse cardiac remodelling [46,49,53]. For instance, it was recently reported that CCR2^+^ and CCR2^−^ macrophages associate with cardiomyocytes in a qualitatively different manner [53]. CCR2^−^ macrophages extend processes that contact adjacent cardiomyocytes, whereas CCR2^+^ macrophages extend processes into the interstitial space but do not abut cardiomyocytes [53]. The physical interaction between CCR2^−^ macrophages and cardiomyocytes occurs through focal adhesion complexes [53]. CCR2^−^ macrophages abundantly express the calcium channel protein transient receptor potential vanilloid 4 (TRPV4) and mechanical stretch promotes pro-angiogenic growth factor expression by CCR2^−^ macrophages in a TRPV4-dependent manner, which attenuates adverse remodelling [53]. Whereas CCR2^−^ macrophages are emerging as important mediators of cardiac homeostasis, the current review focuses on the predominantly (albeit not exclusively) pathological effects of CCR2^+^ macrophages.

## 7. C-C Motif Chemokine Ligand 2 (CCL2)-CCR2 Mediated Monocyte Infiltration in Myocardial Injury

Peripheral monocytosis and the trafficking of monocytes to the site of inflammation involve the simultaneous actions of several chemokines and chemokine receptors [40]. Among these, CCL2-CCR2 mediated monocyte chemotaxis plays a central role in the egression of CCR2^+^Ly6Chi monocytes from the bone marrow and their infiltration into the myocardium [54,55]. Briefly, the interaction of CCL2 (also termed monocyte chemoattract protein-1 (MCP-1)) with the cell surface transmembrane receptor, CCR2 coupled to heterotrimeric G proteins, leads to activation of an intracellular signalling cascade that triggers monocyte mobilization towards the chemokine source [54]. CCL2 dimerization and association with tissue glycosaminoglycans help to establish the chemokine gradient necessary to guide the monocytes to the injury site [40]. CCR2-activated intracellular signalling that facilitates chemotaxis involves pathways mediated by Janus kinase 2 (JAK2)/signal transducer and activator of transcription 3 (STAT3), mitogen-activated protein kinase (MAPK) and phosphoinositide 3-kinase (PI3K) [56,57]. This process of CCL2-CCR2-mediated monocyte recruitment from the bone marrow is restricted to Ly6Chi monocytes. Ly6Clo monocyte recruitment is mediated by C-X3-C motif ligand 1 (CX3CL1; also called fractalkine)–CX3CR1 interaction [58].

Myocardial injury imposes a huge demand for monocytes to be at the site of inflammation. This increased demand for monocytes under inflammatory conditions is met by increased hematopoiesis in the bone marrow [34]. In certain circumstances, such as in severe myocardial injury, the requirement for monocytes is such that the bone marrow alone cannot meet the demand, in which case hematopoiesis also takes place at extramedullary sites, such as in the spleen. However, monocyte mobilization from the spleen may not involve CCL2-CCR2 signalling [59]. In the bone marrow, Ly6Chi monocytes are derived from CCR2^+^ hematopoietic stem cells through sequential developmental stages involving common myeloid progenitors (CMP), granulocyte-macrophage progenitors (GMP), common macrophage and dendritic cell (DC) precursors (MDP) and, finally, committed monocyte progenitors (cMoP) [41]. This process has been reported to be regulated by the action of interleukin-1β (IL-1β) released from the myocardium acting on bone marrow stem cells [60].

In the heart, CCL2 is expressed and secreted by multiple cell types, including macrophages, fibroblasts, epithelial and endothelial cells. CCL2 secretion is enhanced in the presence of inflammatory stimuli including IL-1β, tumor necrosis factor-α (TNF-α), transforming growth factor-β (TGF-β), vascular endothelial growth factor (VEGF), platelet-derived growth factor (PDGF), macrophage colony-stimulating factor (M-CSF) and granulocyte-macrophage colony-stimulating factor (GM-CSF) [61]. In addition to the interaction of CCR2 with CCL2, the expression of cell adhesion molecules, including intercellular adhesion molecule 1 (ICAM-1), vascular cell adhesion molecule 1 (VCAM-1), P-selectin and E-selectin on the endothelial surface are also important in facilitating monocyte attachment and the extravasation of monocytes into the myocardium [62]. It is worth emphasizing though that, although a classical monocytosis response occurs following injury and triggers the entry of these cells into tissues, the actual contribution of monocytes to tissue remodelling is largely mediated through their differentiation into macrophages. In the following sections, we summarize some of the key findings in the literature that describe the specific roles of CCR2^+^ macrophages in ventricular remodelling, categorized according to the different types of myocardial injury that were studied. Table 1 provides a summary of these reports.

## 8. Experimental Studies of CCR2^+^ Monocytes and CCR2^+^ Monocyte-Derived Macrophages in Ventricular Remodelling

### 8.1. Myocardial Infarction

Studies of coronary artery ligation have generally reported that the prevention of CCR2^+^ cell recruitment to the injured myocardium attenuates ventricular remodelling. These experiments were initially performed using strategies that block chemokine signalling or by employing mice with genetic knockout of *Ccr2*. For example, the transfection of mice with an N-terminal deletion mutant gene encoding the principal ligand of CCR2, CCL2 attenuated macrophage accumulation, LV remodelling and heart failure after ligation of the left coronary artery [63]. Likewise, following left anterior descending (LAD) artery ligation, *Ccr2*^−/−^ mice exhibited greatly diminished macrophage infiltration into infarcted tissue, with an attenuation in ventricular remodelling reflected by a diminished increase in left ventricular internal diameter at end diastole (LVDD) and an attenuated decline in fractional shortening (FS) [64]. Two years after the publication of that report, the same group of investigators also demonstrated that, after 45 min ischemia followed by reperfusion, *Ccr2*^−/−^ mice exhibited diminished macrophage infiltration in ischemic lesions and reduced infarct size in comparison to their wildtype counterparts [65]. In 2007, Nahrendorf, Swirski and co-workers reported that the recruitment of Ly6Chi monocytes to myocardial infarcts was decreased by more than 50-fold in *Ccr2*^−/−^ mice, affirming these earlier observations [42]. Interestingly, monocyte recruitment seems to be important in both determining infarct size and determining remodelling of the remote myocardium after MI. In fact, macrophage number in the remote myocardium is higher than in the mature infarct scar [52]. Furthermore, once they are resident in the myocardium, CCR2^+^ macrophages are able to proliferate [52], which may amplify the deleterious actions of these cells in a feed-forward capacity. For example, CCR2^+^ macrophage proliferation in the remodelling ventricle may be induced by increased LV wall tension. Indeed, the exposure of macrophages to biaxial mechanical strain has been reported to induce cellular proliferation accompanied by MAPK pathway activation, whereas mitogen-activated protein kinase kinase (Mek)-1/2 inhibition attenuated strain-induced macrophage proliferation in both cultured cells and the failing myocardium [52].

Various different interventions have been employed to reduce CCR2^+^ monocyte recruitment to the acutely injured heart in MI. For example, Dong and co-workers transplanted hematopoietic stem cells with a lentivirus encoding short interfering RNA (siRNA) directed against hypoxia inducible factor-1α (HIF-1α) into mice prior to LAD ligation [66]. In those experiments, the investigators observed that EF at seven days was significantly higher in mice with hematopoietic stem cell HIF-1α knockdown than in control mice, accompanied by decreased leukocyte CCR1, CCR2 and CCR4 expression [66]. Furthermore, in a chemotaxis assay, decreased monocyte migration induced by HIF-1α downregulation was attenuated by CCR2 overexpression [66]. Majmudar and colleagues performed LAD ligation surgeries in atherosclerosis-prone *ApoE*^−/−^ mice and silenced CCR2 using nanoparticle-encapsulated siRNA [67]. In those studies, knockdown of CCR2 reduced the presence of Ly6Chi monocytes in infarcts, decreased inflammatory gene expression and attenuated ventricular remodelling, as reflected by a lower LVEDV and LVESV and higher EF in comparison to those seen in *ApoE*^−/−^ mice with MI and treated with control siRNA [67]. More recently, it has been reported that CCL2/CCR2-mediated migration to the injured heart following MI is dependent on β2-adrenergic receptor (β2AR) expression by leukocytes [68]. Specifically, Grisanti et al. reported that CCR2 expression and migration in response to CCL2 were both abolished in β2AR knockout bone marrow cells, whereas β2AR agonism with salbutamol increased CCR2 expression [68]. Interestingly, these effects were independent of G-protein-dependent signalling but required β-arrestin2 biased β2AR signalling [68]. Wang et al. combined poly(ethylene glycol) (PEG)-distearoylphosphatidylethanolamine (PEG-DSPE) micelles with a poorly water-soluble CCR2 antagonist and administered the micelles to mice on days 2 and 3 after LAD ligation, assessing infarct size and cardiac function on day 12 [69]. Micelles were either non-targeted or targeted to CCR2-expressing cells by being surface-decorated with an anti-CCR2 antibody [69]. Administration of CCR2-targeting micelles resulted in a ~70% reduction in the accumulation of Ly6Chi cells in the heart and an ~1/3 reduction in infarct size, with a numerical, albeit non-significant, improvement in EF and FS [69]. Lastly, interleukin-10 (IL-10)-producing regulatory B cells (Bregs) are a B-cell subset that has been reported to suppress inflammation. Recently, Bregs were identified as being enriched in pericardial adipose tissue and accumulating in the infarcted heart during the resolution of inflammation, whereas the B-cell-specific deletion of IL-10 worsened cardiac function and delayed resolution of inflammation after MI [85]. Following on from this initial report, Jiao and co-workers reported that the adoptive transfer of Bregs decreased infarct size, attenuated interstitial fibrosis and improved cardiac function after MI, associated with a reduction in Ly6Chi monocyte infiltration [70]. These effects were associated with decreased CCR2 expression by monocytes that reduced the mobilization of monocytes to the injured heart and were reversed by IL-10 neutralization [70].

### 8.2. Pressure Overload

The observations that CCR2^+^ monocytes are recruited to the heart in the setting of pressure overload are especially important because they illustrate that monocyte recruitment to the remodelling heart occurs even in the absence of cardiomyocyte necrosis [86]. Pressure overload hypertrophy that can progress from a compensated form to a decompensated form following surgical constriction of the transverse aorta (transverse aortic constriction, TAC) has been widely employed to explore the roles of CCR2^+^ monocytes and macrophages in non-ischemic ventricular remodelling. In general, most studies have reported that CCR2^+^ monocytes are recruited to mouse hearts early (within one week) after TAC and gradually decline in numbers over subsequent weeks, with CCR2^+^ monocyte recruitment contributing to decompensated remodelling. However, this has not been a universal finding, with other studies reporting a later rise in CCR2^+^ monocytes/macrophages or a biphasic pattern of recruitment.

Patel et al. initially reported that pro-inflammatory monocyte and macrophage expansion occurs within one week following TAC, prior to significant LV hypertrophy (LVH) and the development of systolic dysfunction [87]. However, once LVH was established in response to pressure overload, the depletion of mononuclear phagocytes did not affect cardiac remodelling [87]. Subsequently, using gene expression analysis and flow cytometry, the same group reported that Ly6ChiCCR2^+^ monocytes and CCR2^+^ macrophages accumulate in mouse hearts during the first week after TAC [72]. Furthermore, the treatment of mice with the CCR2 antagonist RS-504393 (2mg/kg twice daily) prevented the early increase in CCR2^+^ macrophage accumulation after TAC and attenuated compensated hypertrophy, whereas treatment of mice with RS-504393 for a longer duration (four weeks) attenuated LV dilatation and systolic dysfunction, and either RS-504393 or a neutralizing antibody directed against CCR2 attenuated interstitial fibrosis [72]. Mechanistically, T cell activation plays an important role in the pathogenesis of pressure overload-induced ventricular remodelling [88,89,90] and, in the study by Patel and co-workers, the prevention of CCR2^+^ macrophage accumulation after TAC also reduced CD4+ and CD8+ T cell expansion in the mediastinal lymph nodes that drain the heart [72]. In contrast to these studies, Nemska et al. reported an early increase in *Ccr2* mRNA levels in mouse hearts after TAC (at 3, 7 and 14 days), but they observed no effect on LV mass increase when RS-504393 (5mg/kg) was administered daily after TAC and continued for 14 days [74]. These investigators also performed banding of the suprarenal abdominal aorta in rats and they observed that, amongst the chemokine receptors and ligands studied by Taqman Low Density Array, both *Ccl2* and *Ccr2* expression peaked at three days after surgery, preceding cardiac hypertrophy, and returning to baseline levels by day 14 [71].

Liao and co-workers described a biphasic expansion of CCR2^+^ macrophages in mouse hearts following TAC [73]. In particular, Ly6G^−^/F4/80^+^/CD64^+^ macrophages increased by three days after TAC, peaked at seven days, returned to baseline by two weeks and then increased modestly four weeks after TAC [73]. The investigators concluded that the majority of macrophages accumulating one week after TAC are proliferating resident CCR2^−^ macrophages and that monocyte infiltration after pressure overload is a late event that coincides with the transition from cardiac compensation to decompensation [73]. Correspondingly, in these studies, CCR2 antagonism with RS-504393 (2mg/kg twice daily) did not affect early macrophage accumulation after TAC, whereas *Ccr2* knockout prevented the decline in EF that was observed eight weeks after TAC [73]. This preservation of cardiac function was associated with an improvement in capillary density without affecting cardiac hypertrophy or fibrosis [73]. Likewise, cardiac function was also preserved when RS-504393 treatment was initiated two weeks after TAC [73]. Similarly, in a recent single-cell RNA sequencing analysis of mouse hearts, Ren et al. reported that although immune cell recruitment is an early event following TAC, the activation of proinflammatory macrophages occurs later (at approximately five weeks), coinciding with a decline in EF [91].

More recently, single-cell transcriptomics have been able to more precisely resolve the kinetics of *Ccr2*+ cell recruitment to TAC hearts and more precisely characterize different macrophage subpopulations in remodelling hearts. For example, recently, Martini, Kunderfranco and co-workers used single-cell transcriptomics of CD45+ cells sorted from mouse hearts one and four weeks after TAC to map cardiac immune cell composition in response to pressure overload [47]. They observed that most immune cell populations are present in healthy and diseased mouse hearts (including macrophages, B cells, T cells, Tregs, Natural Killer cells, neutrophils and mast cells) and that immune activation occurs across these cell-types [47]. Two populations of CCR2^−^ macrophages were identified, one that corresponded to reparatory MHC-IIhi (antigen presenting) CCR2^−^ tissue resident macrophages and the other having low levels of MHC-II, possibly representing a resident population of CCR2^−^ macrophages that contributes to tissue homeostasis by phagocytosing dead cardiomyocytes [47]. Two clusters were identified that corresponded with proinflammatory bone marrow-derived monocytes/macrophages, one of which increased at both one and four weeks after TAC, and the other that increased at four weeks after TAC [47]. Revelo et al. used CyTOF to characterize immune cells in sham-operated mice and mice one and four weeks after TAC, identifying all major immune cell-types, with the majority of immune cells being macrophages followed by monocytes, dendritic cells, neutrophils and B cells [75]. These investigators reported that both resident macrophages and monocyte-derived macrophages increased one week after TAC and largely returned to levels seen in sham-operated mice by four weeks [75]. Single-cell RNA sequencing of CD45+ immune cells isolated from sham mice and mice one week after TAC identified 12 clusters of monocytes and macrophages in pressure overloaded mouse hearts, five clusters of T cells and three clusters of B cells [75]. Eight of the macrophage clusters increased in abundance after TAC [75]. Importantly, in that study, macrophage depletion experiments using a blocking antibody against CD115 (macrophage colony-stimulating factor 1), combined with TAC studies in *Ccr2*^−/−^ mice, revealed that monocyte-derived CCR2^+^ macrophages are major promoters of cardiac fibrosis in pressure overload, which is partially counterbalanced by the actions of resident cardiac macrophages [75].

Single-cell transcriptomics have also recently been used to alternatively characterize pro-inflammatory cardiac macrophages. By performing experiments with the TAC and angiotensin II infusion models of pressure overload, together with LAD ligation studies, Ni et al. recently reported that pro-inflammatory CCR2^+^ cardiac macrophages express high levels of CD72, which is best known as being a B cell regulatory protein [76]. Furthermore, pseudo-time trajectory analysis and chromatin immunoprecipitation (ChIP)-sequencing revealed that CD72hi macrophage differentiation is driven by the transcription factor c-Rel (Rel) [76], which is a member of the nuclear factor kappa-light-chain-enhancer of activated B cells (NF-κB) family and is critical for macrophage polarization [92].

Lastly, whereas some studies have examined the effects of directly blocking CCR2^+^ cell recruitment to TAC hearts, others have explored strategies to indirectly prevent CCR2^+^ monocyte recruitment. Gamma-aminobutyric acid subtype A (GABAA) receptors are principally recognized for their role as neurotransmitter receptors in the central nervous system. However, they are also expressed on immune cells and have been shown to affect ventricular remodelling after MI by influencing monocyte and macrophage subpopulations [93]. In a study of mice with TAC-induced pressure overload, Bu et al. observed that the GABAA receptor agonist topiramate worsened ventricular remodelling, characterized by a reduction in EF and FS and increase in LV end-diastolic diameter (LVEDD) and LV end-systolic diameter (LVESD), myocardial hypertrophy and fibrosis [77]. These effects were associated with a late-phase (four weeks) increase in Ly6Chi monocyte mobilization and CCL2-dependent increase in the percentage of CCR2^+^ macrophages in TAC hearts [77]. Conversely, the GABAA receptor antagonist bicuculline improved ventricular remodelling after TAC [77].

Although not a universal finding [72], some studies in TAC hearts have suggested that CCR2^+^ monocytes and CCR2^+^ monocyte-derived macrophages do not contribute to LVH development, but they may contribute to decompensated remodelling processes such as inflammation, impaired angiogenesis and fibrosis, manifesting as reductions in EF and FS [73,74]. Similar observations have also been made when pressure overload has been induced by infusing mice with the vasoactive peptide, angiotensin II. For example, in an early study, Ishibashi and co-workers reported that CCR2 expression on monocytes increased with angiotensin II infusion, peaking at seven days and being sustained for at least 28 days [78]. This upregulation was prevented by treatment with the angiotensin II type 1 (AT1) receptor blocker (ARB) olmesartan, or in AT1 receptor (AT1R) knockout mice, bone marrow transferred (BMT)-AT1R knockout mice or transgenic mice overexpressing the antioxidant enzyme, superoxide dismutase [78]. Furthermore, whereas angiotensin II-induced vascular inflammation and aortic wall thickening and fibrosis were blunted in *Ccr2*^−/−^ mice and BMT-*Ccr2*^−/−^ mice, LVH was unaffected [78]. Likewise, Xu et al. reported that *Ccr2*^−/−^ mice infused with angiotensin II exhibited a comparable increase in blood pressure and comparable cardiac hypertrophy to their wildtype counterparts, whereas interstitial and perivascular fibrosis were reduced with *Ccr2* knockout [79].

### 8.3. Diabetes

Although diabetes is a common cause of ventricular remodelling in humans [94], comparatively few studies have explored the role or CCR2^+^ macrophages in the diabetic heart. This may be, at least in part, due to the inability of mouse models of diabetes to recapitulate the complex, indolent and heterogeneous nature of heart failure in humans. One study in 2019 did, however, explore the effects of *Ccr2* knockout on heart structure and function in diabetic mice [80]. In that study, Tan et al. observed that CCR2 expression was increased in mouse hearts after 24 weeks of streptozotocin (STZ)-induced diabetes [80]. Furthermore, the investigators reported that STZ-induced diabetes was associated with a reduction in EF, FS and dP/dtmax and an increase in interstitial and perivascular fibrosis, programmed cell death and oxidative stress, whereas these changes were attenuated in *Ccr2*^−/−^ mice [80]. Treatment with the CCR2 antagonist INCB3344 also attenuated the reported reduction in EF, FS and cardiac output (CO) in Type 2 diabetic *db*/*db* mice [80]. It is of note, however, that it is hard to disentangle the cardiac effects of CCR2 knockout or antagonism in these studies of diabetes from their effects on glucose homeostasis [80]. STZ-diabetic wildtype mice, for example, had a greater impairment of glucose tolerance and raised triglycerides in comparison to STZ-diabetic *Ccr2*^−/−^ mice and fasting plasma glucose levels were lower in CCR2 antagonist-treated *db*/*db* mice in comparison to vehicle-treated *db*/*db* mice [80].

### 8.4. Myocarditis

Being viral or autoimmune in etiology, it is not surprising that myocarditis has been reported to induce an inflammatory response that is characterized by the accumulation of pro-inflammatory CCR2^+^ monocytes and macrophages in affected hearts. In 2005, for example, Goser and co-workers explored the roles of CCL2/CCR2 in experimental autoimmune myocarditis (EAM), observing that either CCL2 neutralization or *Ccr2* knockout attenuated myocarditis in BALB/c mice injected with cardiac myosin [81]. A decade later, Leuschner and co-workers reported an ~10-fold increase in CCR2^+^ cells in LV tissue from humans with myocarditis, accompanied by an ~5-fold increase in *Ccl2* and *Ccr2* mRNA levels [51]. The investigators induced myocarditis in mice by subcutaneous injection of Troponin I peptide and in vivo administration of nanoparticle encapsulated siRNA targeting CCR2 resulted in a 69% reduction in Ly6Chi monocytes in inflamed hearts [51]. Interestingly, in that study delayed administration of CCR2 siRNA, initiated 14 days after induction of EAM, attenuated cardiac inflammation and fibrosis and preserved cardiac function at 60 days [51]. Viral myocarditis models have also been used to explore the origin of CCR2^+^ cells in inflamed hearts. For instance, CD45 is a common antigen present on all leukocytes that exists as two functionally identical alleles, CD45.1 and CD45.2. By transferring the bone marrow from mice with one allele to mice with the other it is possible to track the origin of stem cells, progenitor cells and differentiated leukocytes. Lu et al. took this approach and isolated CD45.2^+^CCR2^+^ monocytes/macrophages from the spleen of coxsackievirus B3 (CVB3)-infected CD45.2 mice and injected the cells into CD45.1 mice, detecting CD45.2^+^CCR2^+^CX3CR1^+^ macrophages in the spleen and heart after 48 h and concluding that accumulating CCR2^+^ cardiac macrophages arise from systemic recruitment rather than by local proliferation [82].

### 8.5. Diphtheria Toxin

Diphtheria toxin (DT) models of genetic cardiomyocyte ablation, together with transplantation models of ischemia reperfusion injury (IRI), have been used in meticulous experiments by investigators to determine the origin of CCR2^+^ cardiac immune cells and to determine how these cells mediate their pro-inflammatory effects. In 2014, Lavine et al. employed a diphtheria toxin receptor (DTR) model in which myosin light chain 2v (Mlc2v)-CreRosa26-DTR mice express the DTR specifically in ventricular cardiomyocytes and are susceptible to cardiomyocyte death when administered with DT [45]. In those studies, the investigators observed that neonatal mouse hearts respond to injury by the expansion of MHC-IIloCCR2^−^ macrophages, whereas adult mouse hearts selectively recruit monocytes and MHC-IIhiCCR2^+^ monocyte-derived macrophages [45]. Furthermore, treatment with the CCR2 antagonist RS-504393 (2mg/kg/day twice daily) blocked monocyte recruitment to injured adult hearts, attenuated inflammation and preserved microvascular density [45]. Lavine’s group have subsequently gone on to develop a similar model of cardiomyocyte ablation in which the DTR is expressed under the control of the rat troponin T2 promoter [83]. This model, together with a closed chest IRI approach, was used by the investigators to explore the imaging utility of a positron emission tomography radiotracer (termed 68Ga-DOTA ((1,4,7,10-tetraazacyclododecane-1,4,7,10-tetraacetic acid)-ECL1i (extracellular loop 1 inverso)) that allosterically binds to CCR2 [83]. Whereas the radiotracer was quickly cleared by naïve mice, strong myocardial uptake of the radiotracer was observed in the DT cardiomyocyte ablation model and the closed-chest IRI model that was associated with CCR2^+^ monocyte/macrophage infiltration and that was absent in *Ccr2*^−/−^ mice [83]. Furthermore, autoradiography demonstrated that the radiotracer also binds to human heart failure tissue with signal intensity correlating with CCR2^+^CD68^+^ cell abundance [83]. Thus, DT models have proven useful in both defining the in vivo actions of CCR2^+^ monocytes/macrophages and developing new tools with which to track these cells.

### 8.6. Ischemia Reperfusion Injury (IRI) and Cardiac Transplantation

Studies of CCR2^+^ cardiac immune cells in IRI have been performed using closed chest models of repetitive ischemia or alternatively syngeneic models of cardiac transplantation. In 2007, using a closed-chest model of repetitive 15 min coronary occlusions, without tissue infarction, investigators observed that either genetic knockout of *Ccl2* or CCL2 neutralization diminished interstitial fibrosis and attenuated LV dysfunction [84]. The transplantation model has more recently been used to gain insights into how CCR2^+^ cardiac monocyte-derived macrophages augment cardiac inflammation. One mechanism by which this occurs is through the recruitment of other pro-inflammatory leukocytes, including neutrophils. This mechanism was neatly documented by Li and co-workers, who employed intravital 2-photon imaging to track neutrophils in transplanted mouse hearts. Syngeneic transplantation enables investigators to distinguish the role of resident immune cells from recruited immune cell populations, the former being derived from the donor and the latter being derived from the recipient [50]. In this model system, neutrophil recruitment to donor hearts was diminished when CCR2^+^ monocytes and macrophages were depleted by DT from donor CCR2-DTR mouse hearts [50]. Mechanistically, the investigators went on to discover that neutrophil recruitment by tissue resident CCR2^+^ macrophages was dependent on signalling by MyD88 in cardiac macrophages, which functions as an adaptor protein to mediate toll-like receptor signalling and regulate the expression of neutrophil chemokines [50]. Most notable amongst these chemokines were CXCL2 and CXCL5, which facilitated the transendothelial migration of neutrophils into injured hearts [50]. Interestingly, in a follow-up study, the same group of investigators reported that tissue resident CCR2^+^ cardiac macrophages also facilitate the recruitment of monocytes to the injured heart and their subsequent differentiation into inflammatory monocyte-derived macrophages [49]. Through studying mice with MI, mice with reperfused MI (IRI) and mice with DT-induced cardiomyocyte ablation, Bajpai et al. confirmed that, after myocardial injury, resident macrophages are largely replaced by CCR2^+^Ly6Chi monocytes and CCR2^+^ monocyte-derived macrophages [49]. Furthermore, by combining syngeneic cardiac transplantation to model IRI and intravital 2-photon microscopy, the investigators discovered that tissue-resident CCR2^+^ cardiac macrophages promote monocyte recruitment through the MyD88-dependent release of monocyte chemoattractant proteins (regulating monocyte release and recruitment) [49]. This mechanism is qualitatively distinct from that by which neutrophils are recruited, which depends on the promotion of neutrophil adhesion to the endothelium and transendothelial migration [50].

## 9. CCR2^+^ Cardiac Macrophages in Human Heart Failure

As outlined in the preceding sections, most of the data linking CCR2^+^ macrophages to adverse ventricular remodelling were derived from experimental mouse models. Recent evidence, however, suggests that similar mechanisms are also at play in human heart failure, as elegantly set out in a seminal study by Bajpai and co-workers [48]. In that study, flow cytometry was first performed on LV myocardial specimens from patients with dilated cardiomyopathy (DCM) or ischemic cardiomyopathy (ICM), obtained at the time of left ventricular assist device (LVAD) implantation or heart transplantation [48]. Using this approach, the investigators observed the presence of three distinct macrophage subsets in human failing hearts based on the expression of Human Leukocyte Antigen—DR subtype (HLA-DR; the human homologue of MHC-II) and CCR2 [48]. These populations were CCR2^+^HLA-DRlo, CCR2^+^HLA-DRhi and CCR2^−^HLA-DRhi [48]. Immunostaining revealed that CCR2^−^ macrophages resided close to coronary endothelial cells and CCR2^+^ macrophages were common in sites of scar or tissue fibrosis [48]. To determine the origin of the cardiac macrophages, the investigators studied endomyocardial biopsy specimens from sex-mismatched heart transplant male recipients who received a heart from a female donor, in which Y chromosome positive macrophages in the donor heart were interpreted as originating from recruited male recipient monocytes [48]. Less than 1% of CCR2^−^ macrophages contained a Y chromosome, consistent with these cells being tissue-resident [48]. In contrast, 30% of CCR2^+^ macrophages contained a Y chromosome, suggesting that these cells arose from CCR2^+^ monocyte recruitment [48]. Both cell populations contained a notable number of Ki-67^+^ cells (~10–30%), suggesting that local proliferation contributes to both CCR2^−^ and CCR2^+^ cardiac macrophage populations in human failing hearts [48]. Transcriptomic profiling performed after cell-sorting revealed that CCR2^+^ macrophages were enriched for pro-fibrotic, hypertrophy and inflammatory genes and genes associated with matrix degradation, whereas CCR2^−^ macrophages were enriched for growth factors, extracellular matrix genes and conduction genes [48]. Furthermore, basal and lipopolysaccharide (LPS)-induced expression of the pro-inflammatory mediators IL-1β and chemokine (C-C motif) ligand 7 (CCL7) were higher in CCR2^+^ macrophages than CCR2^−^ macrophages, as was secretion of IL-1β [48]. Lastly, study participants who displayed an improvement in LV function after LVAD implantation had a lower percentage of CCR2^+^ macrophages both before and after LVAD implantation [48]. Similarly, the percentage of CCR2^+^ macrophages correlated inversely with change in EF and positively with LV systolic dimension [48]. Put another way, the abundance of CCR2^+^ macrophages in human heart failure was associated with persistent LV systolic dysfunction and adverse ventricular remodelling following mechanical unloading [48].

## 10. Effects of CCR2^+^ Macrophages on Other Resident Cardiac Cell-Types Contribute to Adverse Cardiac Remodelling

While the aforementioned studies provide compelling evidence that CCR2^+^ macrophages promote adverse ventricular remodelling, the precise mechanisms by which these cells mediate their effects are less well-established. That being said, one of the primary mechanisms is likely to be through the influence of CCR2^+^ macrophages on other cells residing within the remodelling heart. For instance, being antigen-presenting cells, macrophages have the ability to activate the adaptive immune system, particularly the T-cell-mediated immune response, which can aggravate the inflammatory cascade. CD4+ T cells have also been implicated in the transition of cardiac hypertrophy to decompensated heart failure [88]. Hence, CCR2^+^ macrophages may promote the progression to heart failure by facilitating the activation and expansion of T cell populations through the presentation of cardiac antigens [72]. Separately, pattern recognition receptors on the surface of proinflammatory macrophages such as CCR2^+^ macrophages can be activated by damage-associated molecular patterns (DAMPs) inducing signalling cascades involving NF-κB, activator protein 1 (AP1) and interferon regulatory factors (IRFs) that promote the production and secretion of proinflammatory cytokines that amplify myocardial inflammation [95].

Several studies have shown that CCR2^+^ macrophage accumulation promotes myocardial fibrosis and that the prevention of CCR2^+^ macrophage accumulation is associated with decreased myocardial fibrosis [49,72,80]. CCR2^+^ macrophages promote cardiac fibrosis through the production of profibrotic mediators such as TGF-β and osteopontin, which activate cardiac fibroblasts [96]. Separately, it has been reported that, in angiotensin-II-infused mice, macrophages stimulated myofibroblast activation by facilitating IL-6 release from fibroblasts, which induced autocrine fibroblast activation via TGF-β/Smad3 signalling [97]. However, it is worth noting that CCR2^+^ macrophages were not universally reported to promote cardiac fibrosis. For instance, Abe and co-workers recently reported that Ly6ChiCCR2^+^ macrophages that accumulate in the hypoxic regions of TAC hearts inhibit fibrosis through secretion of the cytokine onchostatin M [98].

CCR2^+^ macrophage accumulation has also been associated with decreased microvascular density in hypertrophic hearts [73]. Consistent with these cells having anti-angiogenic effects, conditioned medium from CCR2^+^ macrophages has been reported to inhibit endothelial tube formation [45]. Recently, Alonso-Herranz and co-workers studied the influence of macrophages on endothelial cells in an MI model, where they showed that MI was associated with matrix metalloproteinase-14 (MMP14, also called membrane type 1 matrix metalloproteinase (MT1-MMP)) production by macrophages, and this could activate latent TGF-β, with Smad2-dependent paracrine effects on endothelial cells and promoting the endothelial-to-mesenchymal transition [99].

Cardiomyocytes are ultimately responsible for the pumping action of the heart. Hitscherich et al. reported significant downregulation of cardiac troponin T and sarcoplasmic/endoplasmic reticulum calcium ATPase (Serca2) when embryonic stem cell-derived cardiomyocytes (mES-CM) were exposed to media conditioned by LPS-activated proinflammatory RAW 264.7 macrophages [100]. The authors also observed that mES-CM displayed aberrant Ca^2+^ dynamics when co-cultured with RAW 264.7 cells, irrespective of the activation status of the macrophages [100]. Monnerat and co-workers reported that IL-1β secreted by macrophages can induce arrythmias in diabetic mice by altering cardiomyocyte electrical activity [101]. In that study, IL-1β increased action potential duration, characterized by a reduction in transient outward potassium current and enhanced diastolic Ca^2+^ leak from the sarcoplasmic reticulum [101]. In a separate study, investigators reported that the combination of TNF-α and IL-1β increased the Ca^2+^ leak from the sarcoplasmic reticulum of rat ventricular myocytes, contributing to decreases in both Ca^2+^ transient amplitude and contraction [102]. Fei et al. also studied the role of macrophages in facilitating the development of arrhythmias after MI [103]. In that study, the authors observed that macrophages formed gap junctions with cardiomyocytes in MI border zones and that both expression of the calcium-activated potassium channel KCa3.1 and conductance were elevated in the MI border zone, mainly due to macrophage accumulation [103].

**Figure 2:**
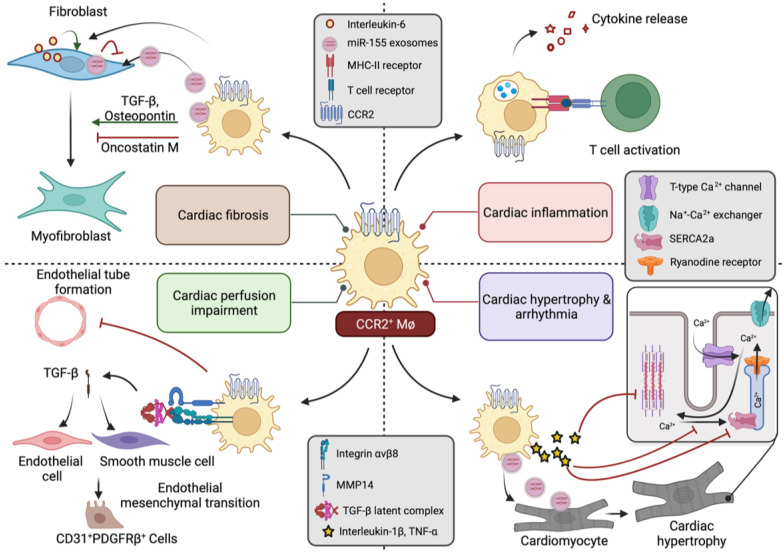
Summarizing some of the reported effects of CCR2^+^ macrophages on other cardiac cell-types. CCR2^+^ macrophages can activate T-cell-mediated immune responses through cardiac antigen presentation. The multiple cytokines secreted by CCR2^+^ cells can also cause cardiac inflammation. CCR2^+^ macrophages can also induce cardiac fibrosis and myofibroblast activation through the secretion of TGF-β and osteopontin. They can also induce fibroblast-mediated IL-6 secretion and autocrine activation of fibroblast proliferation. Other reports have suggested that proinflammatory macrophages can have opposing effects on fibroblasts, for instance, through oncostatin-M-mediated inhibition of myofibroblast activation or through the inhibition of fibroblast proliferation by miR-155-containing exosomes. CCR2^+^ macrophages inhibit endothelial tube formation. They have also been reported to promote endothelial mesenchymal transition through MMP14-mediated release of TGF-β from latent complex. Exosomes released by macrophages containing miR-155 can promote hypertrophy and pyroptosis of cardiomyocytes. Proinflammatory cytokines released by macrophages can also inhibit Ca^2+^ dynamics and affect contractile proteins promoting arrhythmias and impairing contractility. Abbreviations: CCR2 = C-C chemokine receptor type 2, TGF-β = transforming growth factor beta, MMP14 = matrix metallopeptidase 14, PDGFR = platelet-derived growth factor receptor, TNF-α = tumour necrosis factor alpha, SERCA2a = sarco/endoplasmic reticulum Ca^2+^ adenosine triphosphatase-2a.

Lastly, it is worth noting that, whereas the paracrine effects of cardiac macrophages have largely been attributed to increased cytokine production (directly or indirectly), the release of exosome microparticles by macrophages also plays a role. For instance, in one study, microparticles derived from apoptotic RAW 264.7 macrophages were observed to contain soluble TNF-α and impaired cardiomyocyte sarcomere kinetics in a TNF-α dependent manner [104]. Furthermore, in a recent study of uremic cardiomyopathy, it was reported that macrophage-derived miR-155-containing exosomes drove pyroptosis and cardiomyocyte hypertrophy through translational repression of forkhead transcription factors of the O class (FoxO3a) [105]. The effects of CCR2^+^ macrophages on other cardiac cell-types are summarized in Figure 2.

## 11. Unanswered Questions and Future Directions for CCR2^+^ Cardiac Macrophage Research

Although almost two decades have passed since the first recognition of the importance of CCL2/CCR2 signalling in pathological ventricular remodelling [63], recent technological leaps have revealed the heterogeneous nature of cardiac macrophages and they have identified CCR2 as the central gene and cell-surface marker, identifying a subset of cardiac macrophages that functionally contribute to ventricular remodelling. Furthermore, these deleterious effects of CCR2^+^ macrophages, observed in experimental rodents, seem likely to be replicated in the human heart failure setting [48]. In contrast, resident *Ccr2*^−^ macrophages appear to have a counterbalancing, homeostatic function (Table 2). Whereas these advances have shifted the paradigm away from the classical M1/M2 dogma towards a more nuanced appreciation of cardiac macrophage heterogeneity, a number of questions remain unresolved and a number of limitations of the experimental studies warrant consideration.

Most studies that examine the functional effects of CCR2-expressing monocytes and macrophages do so using mice with global knockout of *Ccr2*, neutralization of the CCR2 ligand CCL2, systemic knockdown of CCR2 or systemic administration of a small-molecule CCR2 antagonist. However, CCR2 expression is not limited to monocytes and macrophages but has also been reported to be present on endothelial cells [106], fibroblasts [79], basophils [107] and some T lymphocytes [108]. These global approaches to CCR2 knockdown/knockout or inhibition/antagonism will thus affect other CCR2-expressing cells. The contribution of the functional effects of CCR2 knockout or antagonism in non-myeloid cells (if any) to the cardiac phenotype reported is thus uncertain. This limitation could be circumvented through the use of a cell-specific knockout approach that deletes *Ccr2* solely from monocytes and macrophages. However, current strategies aimed at targeting monocytes and macrophages for cell-specific knockout typically employ a Cre recombinase insertion into the coding region of Lyz2 that is also expressed by neutrophils [109]. Likewise, studies exploring the functional effects of resident CCR2^−^ cardiac macrophages have generally drawn conclusions based on the depletion of CD169^+^ cells rather than specifically CCR2^−^ cardiac macrophages [110]. Furthermore, it has also been proposed that the mobilization of monocytes from the spleen occurs independently of CCR2 [59]. As such, systemic knockout or antagonism of CCR2 may not be expected to prevent monocyte egress from the spleen. That being said, preclinical studies with CCR2 antagonists have demonstrated a significant reduction in the number of Ly6Chi monocytes in the blood and in their infiltration of the remodelling myocardium, indicating the specificity and major involvement of the CCL2/CCR2 axis in this process [72]. CCR2 antagonists are under clinical investigation for other indications, for instance, in kidney disease in diabetes [111]. However, the redundancy of the chemokine receptor system and poor drug-like properties and pharmacokinetic properties of some experimental agents have limited the progress of CCR2 antagonizing therapies into the clinic [112]. In the future, these limitations could be circumvented through the use of antibodies that target multiple chemokine receptors or through the use of combinatorial therapy with multiple receptor antagonists [112].

The cell-surface expression of CCR2 is important for the mobilization of inflammatory monocytes to the injured heart [72]; CCR2^+^ macrophages that promote monocyte recruitment to the injured heart may already be resident in the heart [49,113] and CCR2^+^ inflammatory macrophages may already be resident in failing human hearts [48]. Accordingly, a therapeutic strategy that targets CCR2-mediated chemotaxis itself may not be the most efficacious strategy to attenuate the deleterious actions of cells that may already have gained entry to the remodelling myocardium at time of clinical presentation. Instead, it may be preferable to explore strategies that specifically target resident CCR2^+^ macrophages or recruited CCR2^+^ monocytes and macrophages once they have accumulated in the myocardium. These strategies could, for instance, involve the epigenetic modulation of their inflammatory phenotype [114,115]. Alternatively, they could focus on the mechanisms by which CCR2^+^ macrophages affect other resident heart cells, including cardiomyocytes [52,91]. In this regard, it is noteworthy that several mechanisms have been identified that promote the upregulation of chemokines and subsequent monocyte recruitment to the injured heart, including the activation of neurohumoral signalling pathways, oxidative stress and mechanical strain [86]. However, the downstream pathways in other cells that are activated by CCR2^+^ cardiac macrophages and promote adverse remodelling are comparatively understudied. Similarly, it is unclear to what extent the contribution of CCR2^+^ macrophages to ventricular remodelling is due to the effects of these cells on other resident cardiac cells, and to what extent their effects are mediated by the recruitment of other inflammatory cells. Resident CCR2^+^ macrophages promote the recruitment of both neutrophils and monocytes to the remodelling heart, although the mechanisms underlying this recruitment appears to be qualitatively different for neutrophils and monocytes [49,50]. Furthermore, when monocyte recruitment after MI was blocked with the CCR2 antagonist RS-504393 (2mg/kg/day), investigators observed a diminution in the accumulation and activation of CD4+ T cells in the heart and in the mediastinal lymph nodes that drain the heart [116]. Thus, an alternative strategy to attenuate the pathological effects of CCR2^+^ macrophages could be to prevent their recruitment of other pro-inflammatory immune cells.

Lastly, researchers should remain cognizant that the effects of recruited CCR2^+^ macrophages after myocardial injury are not always detrimental but may also play a necessary reparative role after injury, for instance, by phagocytosing necrotic cardiomyocytes. By way of example, transgenic mice overexpressing CCL2 have been reported to have improved LV dysfunction following IRI [117]. Separately, it has been suggested that the cardioprotective effects of stem cell therapy after IRI are mediated by the induction of an acute sterile immune response, characterized by the induction of CCR2^+^ and CX3CR1^+^ macrophages [118]. Similarly, CCR2^+^ bone marrow cells have been reported to be important for the formation of collateral vessels following LAD ligation [119]. Thus, a blanket therapeutic targeting of CCR2^+^ cell recruitment after myocardial injury is unlikely to result in universally favorable outcomes in ventricular remodelling. Longitudinal studies employing spatial transcriptomics would be helpful in understanding whether disease-specific macro- and micro-specific tissue niches exist and in explaining the complex heterogeneity in macrophage subpopulations and their differential functional patterns [43].

## 12. Conclusions

In summary, CCR2^+^ macrophages play major roles in ventricular remodelling in experimental rodents, and they likely have similar effects in human heart failure. These roles are mediated, at least in part, by the cell-surface expression of CCR2 that promotes monocyte chemotaxis and by the inflammatory phenotype of CCR2^+^ monocyte-derived macrophages. Although these observations would ostensibly identify CCR2^+^ macrophages as promising therapeutic targets for strategies aimed at improving ventricular remodelling, a more nuanced understanding of their function and their effects is required. In some settings, the recruitment of CCR2^+^ bone marrow cells to the injured heart can also have disease-attenuating effects, CCR2^+^ macrophages with pro-inflammatory potential may already be present in normal hearts and CCR2^+^ macrophages may already have accumulated in human heart failure at the time of clinical presentation. In recent years, the study of macrophage heterogeneity has significantly advanced our understanding of cardiac (patho)physiology. To translate these advances to improvements in care for people with, or at risk of developing, heart failure, a greater understanding is needed as to how CCR2^+^ macrophages actually promote ventricular remodelling.

## Figures and Tables

**Table 1:** Experimental studies reporting the roles of CCR2^+^ monocytes/macrophages in cardiac disease associated with ventricular remodelling.

Myocardial Infarction			
Year	Disease Context and Intervention	Principal Effects on Ventricular Remodelling	Citation
2003	Left coronary artery ligation and limb skeletal muscle transfection of N-terminal deletion mutant human CCL2	Mutant CCL2 transfection improved survival, LV cavity dilatation, contractile dysfunction, interstitial fibrosis, macrophage recruitment and inflammatory and fibrotic gene expression	[63]
2004	LAD ligation in wildtype and *Ccr2*^−/−^ mice	*Ccr2* knockout decreased macrophage accumulation, interstitial fibrosis and rise in LVDD and attenuated FS decline	[64]
2006	45 min ischemia reperfusion by occlusion of left coronary artery in wildtype and *Ccr2*^−/−^ mice	Decreased macrophage accumulation, infarct size and oxidative stress with *Ccr2* knockout	[65]
2007	Left coronary artery ligation in wildtype and *ApoE*^−/−^ mice	Sequential recruitment of Ly6C^hi^ and Ly6C^lo^ monocytes to infarcted hearts via CCR2 and CX3CR1, respectively. Impaired wound healing in *ApoE*^−/−^ mice with persistent Ly6C^hi^ monocytosis after MI	[42]
2009	Left coronary artery ligation	Splenic Ly6C^hi^ monocytes are recruited to the ischemic myocardium	[59]
2010	LAD ligation and lentiviral transfection of transplanted hematopoietic stem cells with HIF-1α siRNA	Decreased leukocyte CCR2 expression and improved EF with HIF-1α knockdown	[66]
2013	Knockdown of CCR2 with nanoparticle-encapsulated LAD ligation in wildtype and *ApoE*^−/−^ mice	CCR2 knockdown decreased Ly6C^hi^ monocytes in infarcts, inflammatory gene expression and LVEDV and LVESV and increased EF	[67]
2016	LAD ligation	Macrophage accumulation in the remote myocardium occurs through both local macrophage proliferation and monocyte recruitment	[52]
2016	LAD ligation in bone marrow chimeric β2AR knockout mice or CCR2 knockout mice or treatment with CCR2 antagonist	Leukocyte recruitment to infarcted hearts diminished by β2AR knockout, CCR2 knockout or CCR2 antagonist	[68]
2018	Administration of CCR2-targeting micelles containing CCR2 antagonist to mice after LAD ligation	Decreased Ly6C^hi^ cell accumulation and reduced infarct size with CCR2 antagonism	[69]
2021	LAD ligation and adoptive transfer of Bregs	Decreased infarct size, Ly6C^hi^ monocyte infiltration and interstitial fibrosis, LVEDD and LVESD and increased EF and FS associated with downregulation of monocyte CCR2 expression	[70]
**Pressure Overload**			
**Year**	**Disease Context and Intervention**	**Principal Effects on Ventricular Remodelling**	**Citation**
2016	Banding of the suprarenal abdominal aorta in rats	Upregulation in cardiac *Ccl2* and *Ccr2* mRNA levels preceding LVH	[71]
2018	CCR2 antagonism and antibody-mediated CCR2^+^ monocyte depletion in mice with TAC	CCR2 antagonism prevented early macrophage accumulation and attenuated LVH. Longer duration treatment attenuated both LV dilatation and EF decline. Either CCR2 antagonism or anti-CCR2 antibody attenuated interstitial fibrosis	[72]
2018	TAC in wildtype and *Ccr2*^−/−^ mice and CCR2 antagonism	Concluded that macrophage accumulation early after TAC is due to proliferation of resident CCR2^−^ macrophages and monocyte infiltration is a later event. CCR2 antagonism did not affect early macrophage accumulation. *Ccr2* knockout prevented EF decline and preserved capillary density without affecting hypertrophy or fibrosis. Delayed CCR2 antagonism attenuated EF decline and did not affect hypertrophy	[73]
2019	Single-cell RNA sequencing of CD45^+^ cells from mouse TAC hearts	Described four clusters expressing macrophage/monocyte markers: *Ccr2*^−^ pro-repair macrophages, *Ccr2*^−^ phagocytic monocytes/macrophages and two *Ccr2*^+^ proinflammatory recruited populations	[47]
2021	TAC in wildtype mice	*Ccr2* mRNA levels increased in mouse hearts 3–14 days after TAC and CCR2 antagonism did not affect LVH	[74]
2021	CyTOF and single-cell RNA sequencing in wildtype TAC hearts. Antibody-based macrophage depletion. *Ccr2* knockout in TAC mice	Reported that both resident macrophages and monocyte-derived macrophages increased one week after TAC and declined by four weeks. Monocyte-derived CCR2^+^ macrophages are major promoters of cardiac fibrosis	[75]
2021	TAC, angiotensin II and LAD ligation. Rel knockdown and *Rel*^−/−^ bone marrow chimera mice	Pro-inflammatory CCR2^+^ macrophages express high levels of CD72. CD72^hi^ macrophage differentiation is driven by c-Rel. Rel knockout prevented EF decline in TAC mice	[76]
2021	GABAA receptor agonist and antagonist administration to TAC mice	GABAA receptor agonism increased CCR2^+^ macrophage accumulation, LVEDD, LVESD, hypertrophy and fibrosis and decreased EF and FS. GABAA receptor antagonism improved remodelling	[77]
2004	Angiotensin II infusion in wildtype and *Ccr2*^−/−^ and bone marrow transferred *Ccr2*^−/−^ mice	Angiotensin II increased monocyte CCR2 expression. *Ccr2*^−/−^ and bone marrow transferred *Ccr2*^−/−^ mice exhibited blunted aortic remodelling with angiotensin II, whereas LVH was unaffected	[78]
2011	Angiotensin II infusion in wildtype and *Ccr2*^−/−^ mice	Angiotensin II increased blood pressure and LVH comparably in wildtype and *Ccr2*^−/−^ mice, whereas interstitial and perivascular fibrosis were reduced with *Ccr2* knockout	[79]
**Diabetes**			
**Year**	**Disease Context and Intervention**	**Principal Effects on Ventricular Remodelling**	**Citation**
2019	Streptozotocin-induced diabetes in wildtype and *Ccr2*^−/−^ mice. CCR2 antagonism in *db*/*db* mice	*Ccr2* knockout attenuated reduction in EF, FS, dP/dt_max_, fibrosis, programmed cell death and oxidative stress in streptozotocin-diabetic mice. CCR2 antagonism attenuated reduction in EF, FS and CO in *db*/*db* mice	[80]
**Myocarditis**			
**Year**	**Disease Context and Intervention**	**Principal Effects on Ventricular Remodelling**	**Citation**
2005	CCL2 neutralization or *Ccr2* knockout in EAM (cardiac myosin induced). Transfection with dominant negative inhibitor of *Ccl2* gene	CCL2 neutralization, *Ccr2* knockout or dominant negative *Ccl2* transfection attenuated myocarditis severity	[81]
2015	Nanoparticle encapsulated CCR2 siRNA administration to mice with EAM (Troponin I induced)	Attenuated Ly6C^hi^ monocyte recruitment, cardiac inflammation and fibrosis and preserved EF	[51]
2020	EAM (MyHCα_614–629_) and viral myocarditis (CVB3)	Transfer of splenic CD45.2^+^CCR2^+^ monocytes/macrophages to CD45.1 mice showed CD45.2^+^CCR2^+^CX3CR1^+^ macrophages in the hearts 48h after CVB3 infection	[82]
**Diphtheria Toxin**			
**Year**	**Disease Context and Intervention**	**Principal Effects on Ventricular Remodelling**	**Citation**
2014	DT administration to mice expressing DTR in cardiomyocytes (Mlc2v-CreRosa26-DTR) and CCR2 antagonism	Adult mouse hearts selectively recruit MHC-II^hi^CCR2^+^ monocyte-derived macrophages in response to cardiomyocyte death. CCR2 antagonism blocked monocyte recruitment, attenuated inflammation and preserved microvascular density	[45]
2019	DT administration to mice expressing DTR under the control of the rat *Tnnt2* promoter, closed chest IRI and injection of PET ^68^Ga-DOTA-ECL1i radiotracer	^68^Ga-DOTA-ECL1i uptake was associated with accumulation of CCR2^+^ monocytes and macrophages in injured hearts	[83]
**IRI and Cardiac Transplantation**			
**Year**	**Disease Context and Intervention**	**Principal Effects on Ventricular Remodelling**	**Citation**
2007	*Ccl2* knockout and antibody-mediated CCL2 neutralization in mice with closed chest IRI	CCL2 knockout or downregulation decreased monocyte infiltration, fibrosis and FS decline	[84]
2016	Transplantation-associated IRI and intravital 2-photon imaging and depletion of CCR2^+^ cells from donor hearts by DT administration to CCR2-DTR transgenic mice	Tissue resident CCR2^+^ monocyte-derived macrophages mediate neutrophil recruitment to the ischemic myocardium	[50]
2019	MI, reperfused MI, DT/DTR (*Tnnt2* promoter) cardiomyocyte ablation and cardiac transplantation with intravital 2-photon imaging	Tissue resident CCR2^+^ macrophages promote monocyte recruitment and tissue resident CCR2^−^ macrophages inhibit monocyte recruitment	[49]

Abbreviations: LAD = left anterior descending, LVDD = left ventricular internal diameter at end diastole, FS = fractional shortening, MI = myocardial infarction, EF = ejection fraction, LVEDV = left ventricular end-diastolic volume, LVESV = left ventricular end-systolic volume, β2AR = β2-adrenergic receptor, LVEDD = left ventricular end-diastolic diameter, LVESD = left ventricular end-systolic diameter, TAC = transverse aortic constriction, LVH = left ventricular hypertrophy, CyTOF = cytometry by time of flight, GABAA = Gamma-aminobutyric acid subtype A, CO = cardiac output, CVB3 = coxsackievirus B3, DT = diphtheria toxin, DTR = diphtheria toxin receptor.

**Table 2:** Reported differences between CCR2^−^ and CCR2^+^ cardiac macrophages.

Characteristic	CCR2^−^ Macrophages	CCR2^+^ Macrophages
Known as	Resident macrophages	Infiltrating macrophages (with the exception of a small pool of CCR2^+^ resident macrophages)
Nature	Anti-inflammatory, reparative	Proinflammatory
Ontogeny	Originate during embryogenesis from yolk-sac- and fetal-liver-derived monocyte progenitors	Derived from definitive hematopoietic precursors in the bone marrow and spleen
Replenishment	Self-renewal by in situ proliferation	Proliferation as well as replacement by circulating monocytes
Primary functions	Maintenance of tissue homeostasis, resolution of inflammation and repair of damaged tissue	Inflammation, tissue remodelling after injury/infection, fibrosis
Dynamics of myocardial numbers	Abundantly present in the steady state heart, diminish with myocardial insult	Very low in number under homeostatic conditions but abundantly increase after myocardial injury
Location in heart	Near atrioventricular node, adjacent to endothelial cells and near nerve endings	Near the capillaries and sites of inflammation and injury
Distinguishable surface markers	TIMD4, LYVE-1, SIGLEC-1, CX3CR1	CCR2
Clusters identified based on single cell sequencing	TIMD4 cluster and MHC-II cluster under steady state and disease states	CCR2 and ISG clusters under steady state, expand into multiple clusters under disease conditions
Differentially expressed genes	*Igf1, Hbegf, Bmp2, Cyr61, Pdgfc, Fgf9, Trpv4, CD33,* and *Rhob*	*Il1β, Gdf3, Lgals3, Ccl17, Cxcl19, Itgax, Itgb7, Itgax, Traf1, Tnip3, Tnfsf14, Timp1, Mmp12, Mmp19, Vegfa, Pgf, Col4a1, Col3a1,* and *Fn1*
Pathways enriched for differentially expressed genes	Endocytosis/transport, nervous system development, cell adhesion, and migration	Antigen presentation, immune/inflammatory response, T cell co-stimulation, integrin remodelling and angiogenesis
Effect on monocyte mobilization	Inhibit monocyte infiltration	CCR2^+^ resident macrophages promote monocyte infiltration
Predominant effect on myocardial angiogenesis	Promote angiogenesis	Inhibit angiogenesis
Predominant effect on cardiac fibrosis	Prevent fibrosis	Promote fibrosis
Effect on electrical activity of the heart	Facilitate electrical conduction in the heart through connexin-43-containing gap junctions	Predispose to arrhythmias by increasing duration of action potential
Overall effect on cardiac remodelling and function	Promote healing of the myocardium after injury and restore cardiac function	Promote adverse cardiac remodelling changes resulting in impaired cardiac function
Contact with cardiomyocytes	Foot processes are in direct contact with cardiomyocytes	Not in contact with cardiomyocytes and foot processes extend into interstitial spaces

Abbreviations: TIMD4 = T cell immunoglobulin and mucin-domain-containing 4, LYVE-1 = lymphatic vessel endothelial hyaluronan receptor 1, SIGLEC-1 = sialic acid-binding immunoglobulin-type lectin 1 (CD169), CX3CR1 = C-X3-C Motif Chemokine Receptor 1, CCR2 = C-C chemokine receptor type 2, Igf1 = insulin-like growth factor 1, Hbegf = heparin-binding EGF-like growth factor, Bmp2 = bone morphogenetic protein 2, Cyr61 = cysteine-rich angiogenic inducer 61, Pdgfc = platelet derived growth factor C, Fgf9 = fibroblast growth factor 9, Trpv4 = transient receptor potential vanilloid-type 4, CD33 = sialic-acid-binding Ig-like lectin 3 (Siglec-3), Rhob = Ras homolog family member B, Il1β = interleukin 1 beta, Gdf3 = growth differentiation factor 3, Lgals3 = galectin 3, Ccl17 = C-C motif chemokine ligand 17, Cxcl19 = chemokine (C-X-C motif) ligand 19, Itgax = integrin subunit alpha X, Itgb7 = integrin subunit beta 7, Traf1 = TNF-receptor-associated factor 1, Tnip3 = TNFAIP3-interacting protein 3, Tnfsf14 = TNF superfamily member 14, Timp1 = TIMP metallopeptidase inhibitor 1, Mmp12 = matrix metallopeptidase 12, Mmp19 = matrix metallopeptidase 19, Vegfa = vascular endothelial growth factor A, Pgf = placental growth factor, Col4a1 = collagen type IV alpha 1 chain, Col3a1 = collagen type III alpha 1 chain, Fn1 = fibronectin 1. The reported differences are specific or aggregated findings from the publications included in this review.

## Data Availability

Not applicable.
